# Diosmin Potentiates the Antidiabetic Effects of Linagliptin in Nicotinamide/Streptozotocin-Induced Diabetic Wistar Rats

**DOI:** 10.3390/ph18050656

**Published:** 2025-04-29

**Authors:** Eman B. Abbas, Asmaa M. El-Kalaawy, Noha A. Ahmed, Anwar Shams, Amal K. Khaliefa, Osama M. Ahmed

**Affiliations:** 1 Physiology Division, Department of Zoology, Faculty of Science, Beni-Suef University, P.O. Box 62521, Beni-Suef 62521, Egypt; eman_baker2002@yahoo.com (E.B.A.); osama.ahmed@science.bsu.edu.eg (O.M.A.); 2 Department of Pharmacology, Faculty of Medicine, Beni-Suef University, Beni-Suef 62521, Egypt; asmaa.hussein@med.bsu.edu.eg; 3 Department of Pharmacology, College of Medicine, Taif University, P.O. Box 11099, Taif 21944, Saudi Arabia; 4 Research Center for Health Sciences, Deanship of Graduate Studies and Scientific Research, Taif University, Taif 26432, Saudi Arabia; 5 High Altitude Research Center, Taif University, P.O. Box 11099, Taif 21944, Saudi Arabia; 6 Department of Biochemistry, Faculty of Science, Beni-Suef University, P.O. Box 62521, Beni-Suef 62521, Egypt; bio.biochemistry2011@yahoo.com

**Keywords:** diosmin, linagliptin, nicotinamide, streptozotocin, anti-inflammatory effect, antioxidant effect, diabetic rats

## Abstract

**Background/Objectives:** Natural therapeutics for the treatment of diabetes mellitus represent a common challenge for many researchers. Thus, the aim of this study was to evaluate the antihyperglycemic and anti-inflammatory effects and the hepatic antioxidant activities of both diosmin and linagliptin on nicotinamide/streptozotocin-induced diabetes mellitus in rats. **Methods:** Induction of diabetes mellitus was produced by injecting an intraperitoneal dose of nicotinamide (60 mg/kg) to 16-hour-fasted rats, then after 15 min, an intraperitoneal dose of streptozotocin (60 mg/kg) was injected. The rats with diabetes were orally treated with linagliptin (1 mg/kg), diosmin (10 mg/kg), and both of them every other day for 4 weeks. **Results:** The elevated hepatic glucose-6-phosphatase and glycogen phosphorylase activities, the lowered concentrations of serum insulin, C-peptide, and hepatic glycogen, and the diminished hepatic antioxidant defense system of nicotinamide/streptozotocin-induced diabetic rats were all potentially improved by the therapies. The treatments also improved the deteriorated adiponectin and resistin mRNA expression in visceral adipose tissue of nicotinamide/streptozotocin-induced diabetic rats. In addition, the treatments induced a recovery of damaged islets of Langerhans and a regeneration of islet cells in association with the enhancement of the formation of insulin granules in β-cells and the improvement of kidney function; the combined effect was the most potent. **Conclusions:** Diosmin alone or in combination with linagliptin has potent antidiabetic effects, which were managed through their insulinotropic and insulin-improving actions. The diosmin in combination with linagliptin has the most potent antihyperglycemic effects.

## 1. Introduction

Diabetes mellitus (DM) is a long-term metabolic disease marked by reduced insulin action and/or absolute or relative insulin insufficiency. It is divided into three groups, also known as type 1 DM (T1DM), type 2 DM (T2DM), and other distinct forms of the disease [1]. The number of people living with DM rose from 200 million in 1990 to 830 million in 2022, and DM was the direct cause of 1.6 million deaths and 47% of all deaths in 2021 worldwide, as reported by the World Health Organization (WHO) in 2024 [2].

T1DM is a long-term inflammatory condition caused by the immune system attacking the β-cells in the pancreas that produce insulin [2]. It makes up 5–10% of DM cases, and its occurrence is increasing globally. On the other hand, T2DM accounts for 90% of the frequency of the condition and is far more common [3].

Streptozotocin (STZ) in combination with nicotinamide (NA) causes an animal model to develop a diabetes-like disease that resembles human T2DM [4]. NA is given 15 min before STZ to 2-month-old Wistar rats and causes mild and non-fasting hyperglycemia without a substantial depletion in plasma insulin concentration and a decrease in pancreatic insulin storage, making it a model of impaired insulin sensitivity [5]. STZ administered by intraperitoneal or intravenous routes enters into pancreatic islet β-cells and causes alkylation of the DNA, depletion in the NA dinucleotide, suppression in the cellular adenosine triphosphate, damage to β-cells, and insulin deficiency [6]. Thus, the antagonistic effect of NA causes only modest STZ-induced destruction to pancreatic islets’ β-cells. The results and effectiveness of this two-step technique are determined by the STZ and NA dosages [7]. As a result, STZ/NA-induced animals are an appealing and effective model with pathophysiological parallels to human DM that may be used in acute and chronic research. In this model, pharmacological research of insulinotropic drugs has yielded substances that might be used to treat T2DM [7].

Gliptins, or dipeptidyl peptidase-4 (DPP-4) inhibitors, are among the most recently licensed medications for the treatment of hyperglycemia in T2DM patients. Gliptins exert their antidiabetic benefits principally by blocking the breakdown of endogenous glucagon-like peptide 1 (GLP-1) and glucose-dependent insulinotropic peptide (GIP), hence prolonging postprandial insulin production. DPP-4 inhibitors may have the ability to exhibit a broader range of valuable pleiotropic properties in the heart and vasculature, raising the level of peptides that may have vasoactive and cardioprotective properties. The DPP-4 signaling cascade has recently been shown to play a role in the pathologic characteristics of sepsis, owing to a selective cross-talk within the DPP-4 and nuclear factor-kappa B (NF-κB) signal pathways [8].

Linagliptin is an orally active dipeptidyl peptidase-4 (DPP-4) inhibitor used as T2DM therapy in many countries. This oral, highly specific DPP-4 inhibitor inhibits DPP-4 by more than 80% during a 24 h period. It has nonlinear dose-proportionate pharmacokinetic features and is virtually totally removed through the gastrointestinal system, with less than 5% detected in urine [9].

The primary characteristics of linagliptin are its high efficacy and selectivity for DPP-4 inhibition, as well as its ability to prolong the half-life of circulating hormones and enhance glucose homeostasis in preclinical investigations [9]. In a phase I trial with healthy people, it showed a true 24 h duration of effect and a safety and tolerability profile comparable to a placebo [10].

There is an increasing demand to use natural antidiabetic agents. Because herbal medicines generally have fewer side effects than conventional medications, there has been a resurgence of interest in using them to treat a variety of illnesses in recent years. The World Health Organization (WHO) has also recommended that the effectiveness of plants be evaluated in situations where safe, contemporary medications are lacking. Hence, further assessments of alternative therapies are needed [11].

Diosmin (DS) (diosmetin 7-O-rutinoside), a natural flavanone glycoside, is easily produced by dehydrogenation of hesperidin, which is rich in the pericarp of numerous citrus fruits [12]. Diosmin has anti-inflammatory effects and protects the body from free radicals and other unstable molecules [13]. It is most commonly used to treat hemorrhoids and leg sores caused by inadequate blood flow. It is also said to cure several disorders, although there is no strong evidence to support these claims [14].

Consequently, this study aims to compare the antihyperglycemic, anti-inflammatory, and hepatic antioxidant activities of diosmin and linagliptin in NA/STZ-induced diabetic rats, as well as their interactions. The study also aims to determine whether diosmin enhances linagliptin’s antihyperglycemic effects in diabetic rats.

## 2. Results

### 2.1. Effects on Oral Glucose Tolerance (OGT)

The treatment of diabetic rats with linagliptin, diosmin, and both of them together produced a marked decrease in fasting glucose levels: 242.25 ± 65.8, 366.75 ± 76.49, and 141.25 ± 35.15 mg/dL respectively. The effect was significant (*p* < 0.05) due to treatments with linagliptin and its combination with diosmin. In the first hour of administration of glucose (3 mg/kg), the glucose levels recorded 377.50 ± 88.18, 502.00 ± 65.54, and 255.00 ± 55.6 mg/dL, respectively, reaching their peak value. Then, the glucose levels decreased in the second hour to 287.00 ± 62.09, 405.25 ± 56.05, and 169.75 ± 56.83 mg/dL, respectively. In the third hour, the glucose level recorded 319 ± 76.40 mg/dL in the diabetic rats treated with linagliptin. In the diabetic rats treated with diosmin, the glucose level decreased to 418.67 ± 87.07 mg/dL. The glucose level showed a decrease to 161.25 ± 30.99 mg/dL in the diabetic rats treated with both linagliptin and diosmin. The present data revealed that linagliptin and diosmin together are more effective than treatment with linagliptin alone or diosmin alone (Table 1).

### 2.2. Effects on Serum Insulin and C-Peptide Levels

**Figure 1:**
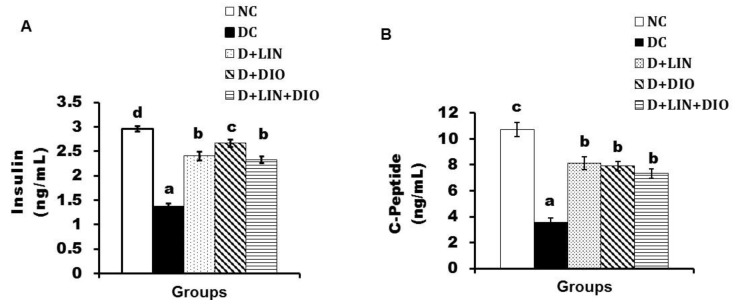
Effects of linagliptin, diosmin, and their combination on the serum insulin level and (**A**) the serum C-peptide level (**B**). Data expressed as the means ± SE. Means which do not share the same superscript symbols (a, b, c, and d) are significantly different at *p* < 0.05. F-probability: *p* < 0.001. NC, DC, D + LIN, D + DIO, and D + LIN + DIO refer to the normal control group, the diabetic control group, the diabetic group treated with linagliptin, the diabetic group treated with diosmin, and the diabetic group treated with linagliptin and diosmin.

Serum insulin level showed a significant depletion (*p* < 0.05) in the diabetic control (1.36 ± 0.07 ng/mL). The treatment with linagliptin, diosmin, and both of them resulted in a significant alleviation of the decreased insulin level to reach 2.41 ± 0.08, 2.67 ± 0.07, and 2.32 ± 0.07 (ng/mL), respectively; the treatment with diosmin was the most effective. Serum C-peptide levels disclosed a significant decrease (*p* < 0.05) in the diabetic rats in comparison with the normal group, reaching 3.56 ± 0.35 (ng/mL). Linagliptin, diosmin, and their combination administered to the diabetic rats recorded 8.12 ± 0.47, 7.90 ± 0.37, and 7.33 ± 0.35 (ng/mL) (Figure 1).

### 2.3. Effect on the HOMA-IR Cell Function, HOMA-IS, and HOMA-β Cell Function

**Figure 2:**
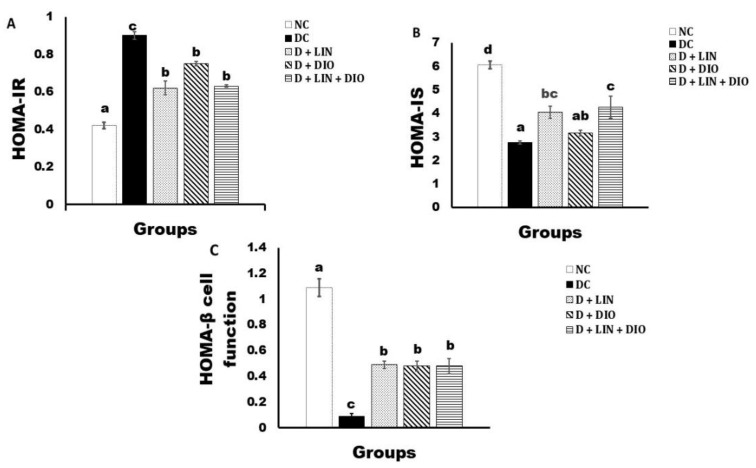
Effects of linagliptin and/or diosmin on the insulin resistance index (**A**), the insulin sensitivity index (**B**), and the β-cell function (**C**). Data expressed as Mean ± SE. Means, which do not share the same superscript symbols (a, b, c, and d) are significantly different at *p* < 0.05. F-probability: *p* < 0.001. NC, DC, D + LIN, D + DIO, and D + LIN + DIO refer to the normal control group, the diabetic control group, the diabetic group treated with linagliptin, the diabetic group treated with diosmin, and the diabetic group treated with linagliptin and diosmin.

HOMA-IR manifested a significant elevation (*p* < 0.05) in the diabetic control in comparison with the healthy rats. The administration of linagliptin, diosmin, and their mixture led to a significant decrease (*p* < 0.05) of the elevated value in the diabetic rats. The HOMA-IS index expressed a significant decrease (*p* < 0.05) in the diabetic group in comparison with the healthy control. The treatment of the diabetic rats with linagliptin, diosmin, and both of them led to a significant increase. The HOMA-β cell function index showed a significant decline (*p* < 0.05) in the diabetic group as compared with the healthy group. The treatment with linagliptin, diosmin, and their mixture resulted in a significant increase (*p* < 0.05) in the diabetic rats (Figure 2).

### 2.4. Effect on the Serum Urea and Creatinine Levels

**Figure 3:**
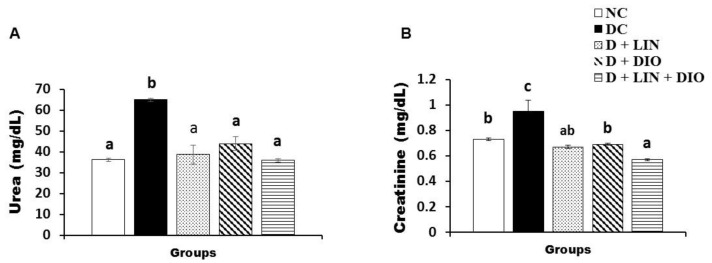
Effects of linagliptin and/or diosmin on the serum urea level (**A**) and creatinine level (**B**). Data expressed as the means ± SE. Means which do not share the same superscript symbols (a, b, and c) are significantly different at *p* < 0.05. F-probability: *p* < 0.001. NC, DC, D + LIN, D + DIO, and D + LIN + DIO refer to the normal control group, the diabetic control group, the diabetic group treated with linagliptin, the diabetic group treated with diosmin, and the diabetic group treated with linagliptin and diosmin.

The serum urea level of the NA/STZ-induced diabetic rats exhibited a significant increase (*p* < 0.05) to 72.7 ± 1.81 mg/dL as compared with the normal group. The serum urea level after treatment with linagliptin recorded 38.72 ± 11.04 mg/dL, while it recorded 43.88 ± 8.06 mg/dL with treatment by diosmin. This level exhibited a significant decrease as a result of the treatment of the diabetic rats with a combination of linagliptin and diosmin together to record 35.9 ± 2.07 mg/dL (Figure 3).

The serum creatinine level of the NA/STZ-induced diabetic rats exhibited a significant increase (*p* < 0.05), recording 0.95 ± 0.09 mg/dl, as compared to the normal group. The serum creatinine level exhibited a significant decrease (*p* < 0.05) when treated with linagliptin, diosmin and their combination, recording 0.67 ± 0.03, 0.69 ± 0.02, and 0.57 ± 0.02 mg/dL respectively. The treatment with a combination of both linagliptin and diosmin was the more effective one (Figure 3).

### 2.5. Effects on Liver Glycogen Content and Glucose-6-Phospatase and Glycogen Phosphorylase Activities

**Figure 4:**
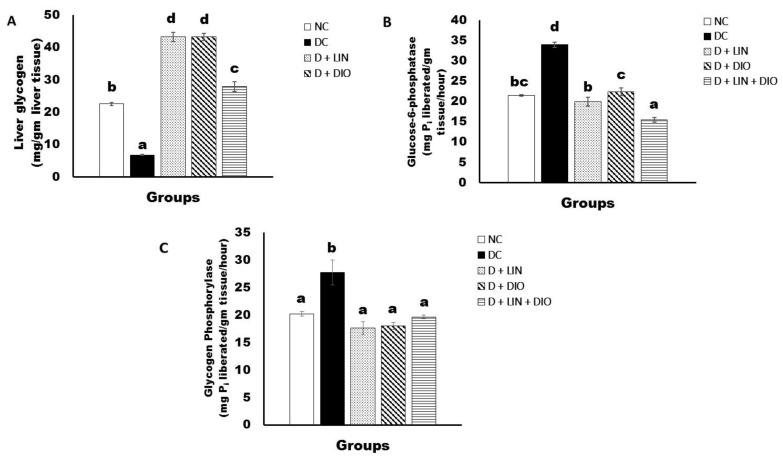
Effects of linagliptin and/or diosmin on the liver glycogen content (**A**), glucose-6-phosphatase activity (**B**), and glycogen phosphorylase activity (**C**). Data expressed as the means ± SE. Means which do not share the same superscript symbols (a, b, c, and d) are significantly different at *p* < 0.05. F-probability: *p* < 0.001. NC, DC, D + LIN, D + DIO, and D + LIN + DIO refer to the normal control group, the diabetic control group, the diabetic group treated with linagliptin, the diabetic group treated with diosmin, and the diabetic group treated with linagliptin and diosmin.

Liver glycogen content of the diabetic rats showed a significant decrease (*p* < 0.05) when compared to the healthy group; the recorded value of the diabetic control was 6.63 ± 0.67 mg/g tissue. The treatment with linagliptin, diosmin, and a combination of them produced a highly significant alleviation (*p* < 0.05) of the liver glycogen content, recording 43.30 ± 1.47, 43.4 ± 1.11, and 27.90 ± 1.48 mg/g tissue, respectively (Figure 4A).

Liver glucose-6-phosphatase activity in the diabetic rats showed a significant elevation (*p* < 0.05) in comparison with the healthy control. The supplementation of linagliptin, diosmin, and their combination to the diabetic rats produced a significant decrease in the serum glucose-6-phosphatase activity, recording 20 ± 1.05, 22.38 ± 0.96, and 15.5 ± 0.61 mg/g tissue, respectively. Thus, the co-administration of linagliptin and diosmin was the most effective treatment (Figure 4B).

Liver glycogen phosphorylase activity in the diabetic rats showed a significant increase (*p* < 0.05) in comparison with the normal group. The treatment with linagliptin, diosmin, and their combination revealed a significant decrease in the liver glycogen phosphorylase, recording 17.62 ± 1.15, 18.07 ± 0.67, and 20.66 ± 0.33 mg/g tissue, respectively (Figure 4C).

### 2.6. Effects on the Liver Oxidative Stress and Anti-Oxidant Defense Parameters

**Figure 5:**
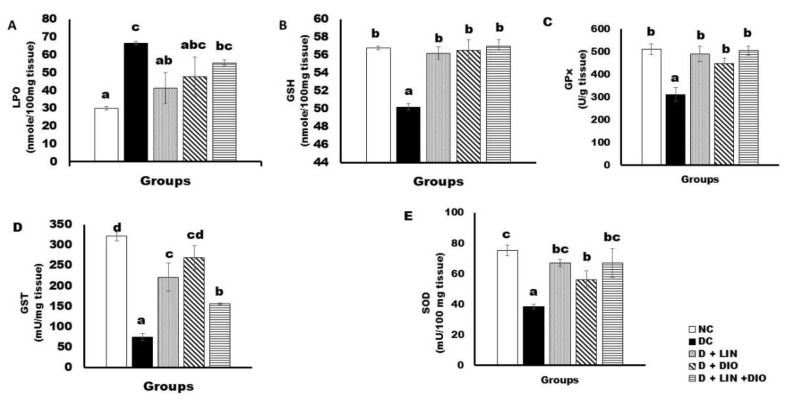
Effects of linagliptin and/or diosmin on LPO (**A**), GSH content (**B**), GPx activity (**C**), GST activity (**D**), and SOD activity (**E**). Data expressed as the means ± SE. Means which do not share the same superscript symbols (a, b, c, and d) are significantly different at *p* < 0.05. F-probability: *p* < 0.001. NC, DC, D + LIN, D + DIO, and D + LIN + DIO refer to the normal control group, the diabetic control group, the diabetic group treated with linagliptin, the diabetic group treated with diosmin, and the diabetic group treated with linagliptin and diosmin.

The data illustrating the effects of treatments with linagliptin, diosmin, and both of them are provided in Figure 5.

The indication of lipid peroxidation (LPO) is the formation of malondialdhyde (MDA). Its level was substantially higher (*p* < 0.05) in the rats with diabetes than in the animals without the disease. Moreover, the treatment with linagliptin resulted in a significant decrease in the MDA level, recording 41.38 ± 8.89 nmol/100 mg tissue. In addition, because of the treatment with diosmin, the MDA level exhibited a significant decrease to 47.78 ± 10.98 nmol/100 mg tissue. The co-administration of linagliptin and diosmin resulted in a decrease of LPO, recording 55.60 ± 1.42 nmol/100 mg tissue (Figure 5A).

The liver reduced glutathione (GSH) content was much lower in the rats with diabetes than in the normal group. Treatment with linagliptin resulted in a significant increase (*p* < 0.05) in the GSH content in the liver, recording 56.20 ± 0.71 nmol/100 mg tissue. Treatment with diosmin as monotherapy exhibited a significant increase in the liver GSH content, recording 56.50 ± 1.19 nmol/100 mg tissue. Under the combination therapy, the level of GSH increased significantly, recording 57.00 ± 0.71 nmol/100 mg tissue (Figure 5B).

The liver glutathione peroxidase (GPx) activity significantly decreased (*p* < 0.05) in the diabetic rats compared with normal control group; the recorded activity in the diabetic rats was 312.33 ± 29.86 U/g tissue. Treatment with linagliptin resulted in a significant increase in the GPx activity, recording 489.75 ± 33.75 U/g tissue. Diosmin treatment produced a significant increase in the GPx activity, recording 449.25 ± 23.18 U/g tissue. The most effective was the treatment with a combination of linagliptin and diosmin that resulted in an increase to 505.20 ± 20.38 U/g tissue (Figure 5C).

The liver glutathione-S-transferase (GST) activity revealed a significant decrease (*p* < 0.05) in the diabetic rats in comparison with the healthy non-diabetic rats, recording 321.40 ± 10.71 mU/mg tissue. Treatment with linagliptin led to a significant alleviation of the GST activity, recording 221.23 ± 34.73 mU/mg tissue. Moreover, treatment with diosmin produced a significant increase (*p* < 0.05) in the GST activity, recording 268.47 ± 29.38 mU/mg tissue. On treatment with a combination of linagliptin and diosmin, a significant increase in the GST activity was recorded, reaching 155.80 ± 1.9 mU/mg tissue (Figure 5D).

The superoxide dismutase (SOD) activity showed a notable reduction in the diabetic rats compared with the normal group, recording 38.50 ± 1.55 mU/100 mg tissue. On treatment with linagliptin, the SOD activity significantly increased, recording 67.00 ± 2.48 mU/100 mg tissue. Treatment with diosmin revealed a significant increase in the SOD activity, recording 56.25 ± 5.66 mU/100 mg tissue. The combination therapy of both treatments caused a significant increase in the SOD activity in the liver, recording 67.00 ± 9.23 mU/100 mg tissue (Figure 5E).

### 2.7. Effect on the Interleukin 10 (IL-10) Level

**Figure 6:**
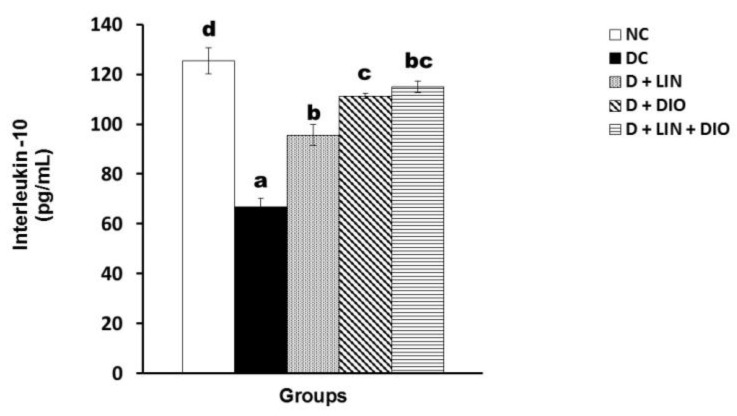
Effects of linagliptin and/or diosmin on the IL-10 level in the serum of the NA/STZ-induced diabetic rats. Data expressed as the means ± SE. Means which do not share the same superscript symbols (a, b, c, and d) are significantly different at *p* < 0.05. F-probability: *p* < 0.001. NC, DC, D + LIN, D + DIO, and D + LIN + DIO refer to the normal control group, the diabetic control group, the diabetic group treated with linagliptin, the diabetic group treated with diosmin, and the diabetic group treated with linagliptin and diosmin.

The IL-10 level is defined as an anti-inflammatory marker. It exhibited a significant decrease (*p* < 0.05) in the serum of the diabetic rats in comparison with the healthy non-diabetic rats, recording 66.87 ± 3.27 pg/mL. Linagliptin treatment effect on the IL-10 level led to a significant increase, recording 95.63 ± 4.19 pg/mL. On diosmin treatment, a significant increase in the level of IL-10 was also observed, recording 111.23 ± 1.03 pg/mL. Treatment with both linagliptin and diosmin together demonstrated a notable increase in the IL-10 level, recording 114.97 ± 2.27 pg/mL (Figure 6).

### 2.8. Effect on the mRNA Expression of Adiponectin and Resistin in Visceral Adipose Tissues

**Figure 7:**
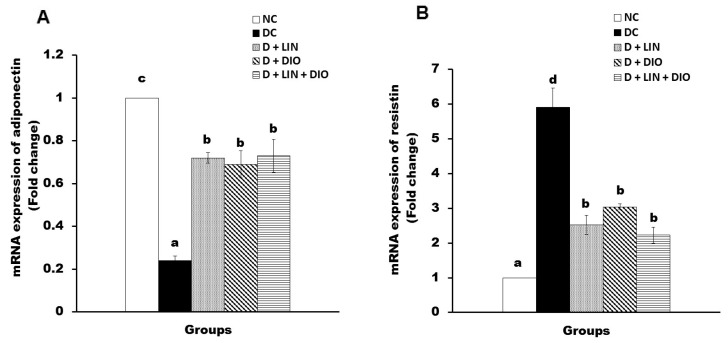
Effects of linagliptin and/or diosmin on the mRNA expression of adiponectin (**A**) and resistin (**B**) in visceral adipose tissue. Data expressed as the means ± SE. Means which do not share the same superscript symbols (a, b, c, and d) are significantly different at *p* < 0.05. F-probability: *p* < 0.001. NC, DC, D + LIN, D + DIO, and D + LIN + DIO refer to the normal control group, the diabetic control group, the diabetic group treated with linagliptin, the diabetic group treated with diosmin, and the diabetic group treated with linagliptin and diosmin.

The diabetic rats showed a significant decrease (*p* < 0.05) in the mRNA expression of adiponectin in visceral adipose tissue. On the other hand, they revealed a significant elevation in the mRNA expression of resistin as compared with the healthy non-diabetic rats. The treatment of the diabetic rats with linagliptin induced a significant enhancement (*p* < 0.05) in adiponectin and a significant alleviation in the mRNA expression of resistin. On treatment with diosmin, the mRNA expression of adiponectin exhibited a significant upregulation while the expression of resistin revealed a significant downregulation. The treatment with both linagliptin and diosmin together exhibited a significant upregulation in the mRNA expression of adiponectin and a significant downregulation in the mRNA expression of resistin; the combined effect was the most potent (Figure 7).

### 2.9. Effect on Pancreatic Histological Changes

**Figure 8:**
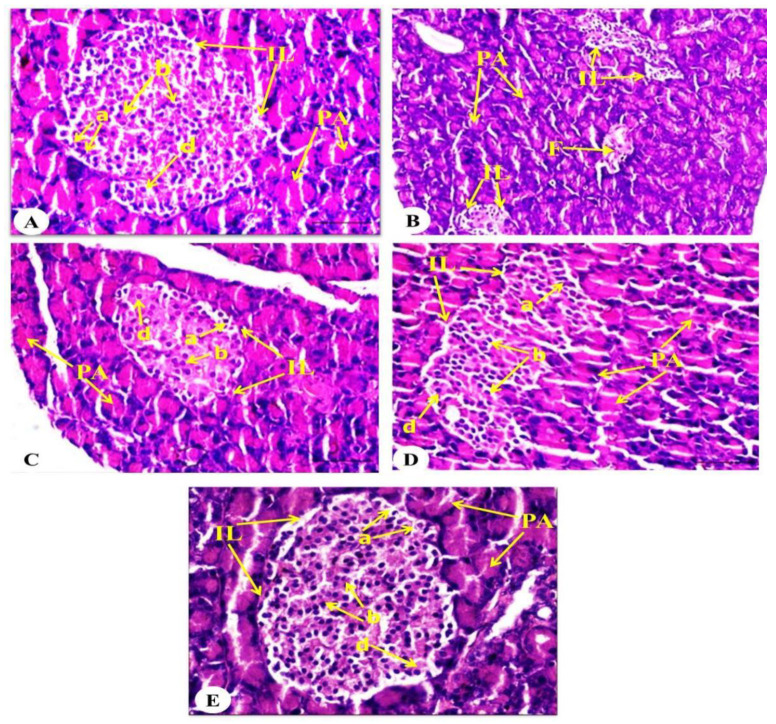
Photographs of H&E stained sections of the pancreas of the normal (**A**), diabetic control (**B**), and diabetic groups treated with linagliptin (**C**), diosmin (**D**), and linagliptin and diosmin (**E**). (**A**) Photograph of a pancreatic section of a healthy rat revealed normal pancreatic acini (PA) and islets of Langerhans (IL) with normal intact α-cells (a), β-cells (b), and δ-cells (d). (**B**) Photograph of a pancreatic section of a diabetic rat revealed small sizes of islets of Langerhans (IL) and fibrosis (F). The islets exhibited an enormous decrease in the islet cells. (**C**) Photograph of a pancreatic section of a diabetic rat treated with linagliptin revealed a marked improvement in the islets of Langerhans (IL) with an increased number of islet cells, including α-cells (a), β-cells (b), and δ-cells (d). (**D**) Photograph of a pancreatic section of a diabetic rat treated with diosmin revealed a remarkable amelioration in the islets of Langerhans (IL), with an increased number of α-cells (a), β-cells (b), and δ-cells (d). (**E**) Photograph of a pancreatic section of a diabetic rat treated with linagliptin and diosmin as combination therapy showed return to normal pancreatic architecture, regenerated islets of Langerhans (IL). The islets had normal integrity, with an increased number of normal and intact α-cells (a), β-cells (b), and δ-cells (d). Magnification for photomicrographs (**A**,**C**–**E**) was 400×, while magnification for photomicrograph (**B**) was 200×.

Figure 8 shows the pancreatic histological alterations in the diabetes control and diabetic treatment groups compared to the normal control. The pancreas is composed of an endocrine portion represented by the islets of Langerhans, which have α-cells at their periphery, β-cells in their core, and larger δ-cells. The exocrine portion is represented by pancreatic acini. The pancreases of the normal rats depicted normal architecture of pancreatic acini and islets with intact α-cells, β-cells, and δ-cells (Figure 8A). The diabetic rats, on the other hand, featured a marked decline of the islets’ size and a decrease in the islet cell number in addition to fibrosis (Figure 8B). The pancreases of the diabetic rats treated with linagliptin (Figure 8C), diosmin (Figure 8D), and linagliptin and diosmin (Figure 8E) exhibited a recovery of damaged islets of Langerhans and regenerated islets. The diabetic rats treated with linagliptin revealed a moderate regeneration of islets of Langerhans. The diabetic rats treated with diosmin revealed a greater increase in the islet cells than those treated with linagliptin. The diabetic rats treated with both linagliptin and diosmin showed a nearly normal pancreatic acini and regeneration of islets of Langerhans. The combined effect seemed to be the most potent.

### 2.10. Effect on the Expression of Insulin in Pancreatic Islets (Immunohistochemical Investigations)

**Figure 9:**
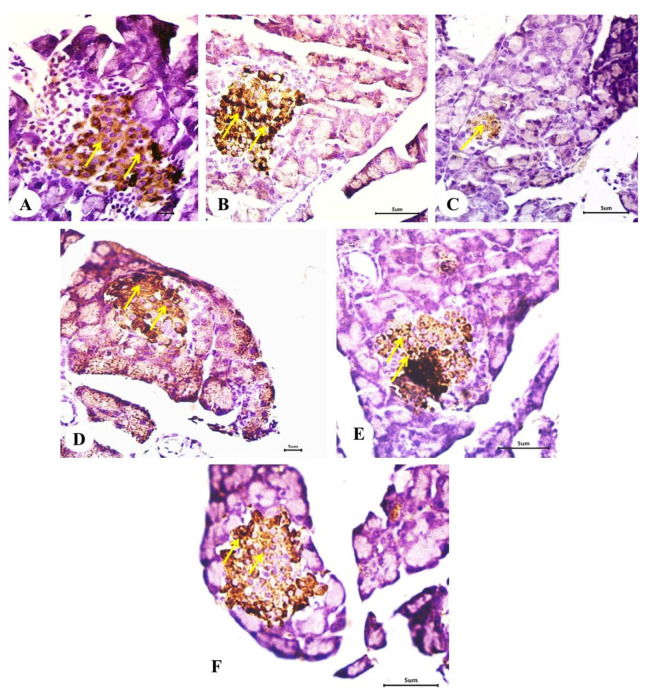
Photomicrographs of immunohistochemically stained sections of the pancreas showing insulin granules in the pancreatic islets of the normal (**A**,**B**), diabetic control (**C**), and diabetic groups treated with linagliptin (**D**), diosmin (**E**), and linagliptin, and diosmin (**F**). (Scale bar: 5 μm). The diabetic rats exhibited a weak expression of insulin when compared with the normal control. On the other hand, the diabetic rats treated with linagliptin and/or diosmin showed a strong expression of insulin granules; the combined effects of linagliptin and diosmin were the most potent. Arrows refer to brown color immunostaining.

**Figure 10:**
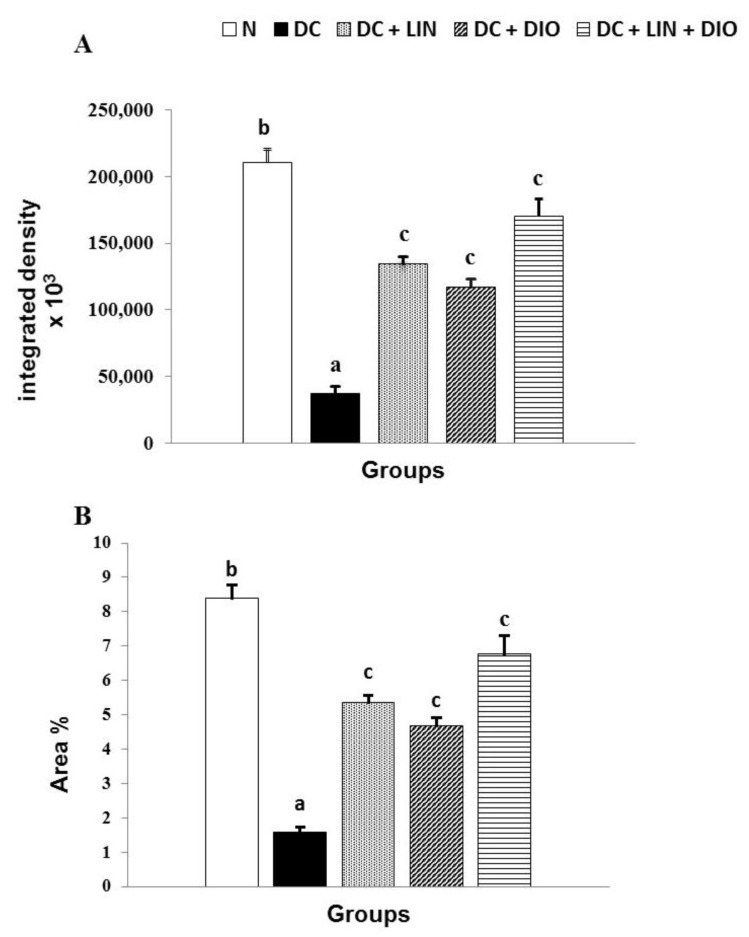
Effects of linagliptin and/or diosmin on the integrated density (**A**) and area percentage (**B**) of brown color produced by immunohistochemical staining of insulin granules of the pancreatic islets in the diabetic rats. Data expressed as the means ± SE. Means which do not share the same superscript symbols (a, b, and c) are significantly different at *p* < 0.05. F-probability: *p* < 0.001. NC, DC, D + LIN, D + DIO, and D + LIN + DIO refer to the normal control group, the diabetic control group, the diabetic group treated with linagliptin, the diabetic group treated with diosmin, and the diabetic group treated with linagliptin and diosmin.

The alterations in the expression of insulin in the pancreatic islets of Langerhans in different groups are depicted in Figure 9A–F. The data obtained from image analysis include density and area percentage of brown color of the immuno-stained islets represented in Figure 10A and Figure 10B, respectively. The diabetic rats exhibited poor immunohistochemical staining (Figure 9C), reflecting a substantial decrease in the concentration of insulin granules as compared with the normal control (Figure 9A,B); the decrease was significant (Figure 10A,B). The treatments of the diabetic rats with linagliptin (Figure 9D), diosmin (Figure 9E), and linagliptin and diosmin (Figure 9F) induced a strong immunohistochemical staining of insulin granules in the pancreatic islets; the effects were significant and the combined effect was the most potent (Figure 10A,B).

## 3. Discussion

A large number of medicinal plants have been used in traditional medicine to treat symptoms of DM, and many of them have undergone some experimental testing for hypoglycemic and hypolipidemic activities [15]. Plant infusions and decoction fluids are widely used by populations in developing and underdeveloped countries since conventional medicines are unavailable or unaffordable. In addition, plant constituents are more preferred than crude extracts to avoid the side effects of other constituents [16,17]. Thus, this study aimed to assess the antihyperglycemic, antioxidant, and anti-inflammatory effects of one plant constituent diosmin alone and in combination with linagliptin in NA/STZ-induced diabetic rats.

STZ plus NA-induced animals with T2DM are an attractive and useful model with pathophysiological similarities to human diabetes, and it is suitable for acute and chronic studies, making it a superior model for evaluating the antihyperglycemic effects of different substances [7].

The oral glucose tolerance test (OGTT) is a valid and widely used assay for determining the antihyperglycemic effects of any hypoglycemic medication [18]. In our study, there was a significant increase in blood glucose levels in the diabetic rats as compared with the normal rats, and this agrees with the results of previous research [19,20,21]. The glucose levels then significantly increased to reach their peak after one hour of administration of glucose. After another hour, the level began to decline until returning to levels close to those detected before oral administration of glucose, to reach normal results in the normal groups and a high level in the diabetic ones. On the other hand, within the treatment group, the level of glucose improved and revealed normal results, especially in the case of combination treatment with both linagliptin and diosmin. This goes parallel with the antihyperglycemic effect of diosmin that was revealed by Ali et al. [22] and agrees with the results of McKeage [23] who stated that linagliptin had an improvement effect on glycemic control in diabetic patients with T2DM when used as monotherapy or in combination with other hypoglycemic agents.

C-peptide is generated during the production of insulin when the precursor molecule called proinsulin is broken down into insulin and C-peptide [24]. It has a consistent metabolic process under a variety of physiological and pathological circumstances [25]. As a result, serum C-peptide is regarded as a more accurate measure of insulin secretion and release than the serum insulin level.

Treatment of the diabetic rats with linagliptin led to a significant increase in both insulin and C-peptide secretion when compared with the diabetic animals which suffer from insulin resistance as well as damage to β-cells in the islets of Langerhans due to the effect of NA/STZ injection. The results of the present study fit with the results of Rauch et al. [26] who indicated that linagliptin was well-tolerated and significantly suppressed the plasma DPP-4 activity in T2DM patients, resulting in immediate enhancements in incretin levels, glucagon suppression, and glycemic management that lasted throughout the study.

Treatment of diabetic animals with diosmin in the present study induced glycemic control in diabetic rats by increasing the serum insulin and C-peptide levels, which may have been due to the elevated number and architecture of β-cells, as detected by histology studies. This agrees with the results of Pari and Srinivasan [27], in which diosmin promoted pancreatic β-cells, which are essential for insulin synthesis and secretion.

In our study, the combination treatment with both linagliptin and diosmin produced improvement of insulin and C-peptide secretion, showing significant glycemic control for diabetic animals by reducing the HOMA-IR and improving both HOMA-IS and HOMA-β-cell function.

In the present study, the liver glycogen content exhibited a significant increase following treatment with linagliptin when compared with the diabetic animals. In contrast with the present study, Shiraki et al. indicated that treatment with linagliptin significantly decreased the glycogen content [28]. In our study, the treatment of diabetic animals with a combination of linagliptin and diosmin revealed a significant increase in the liver glycogen content. The synthesis of hepatic glycogen content was reduced in the diabetic patients due to low insulin levels, which inactivated the glycogen synthase pathway. Thus, the liver glycogen content is a significant marker of glycemic control [28].

The rate-limiting phase in glycogen depletion is catalyzed by glycogen phosphorylase. Thus, its activity increased in the diabetic patients. According to our study, the activity of the glycogen phosphorylase enzyme declined when the diabetic animals were treated with linagliptin [29]. This occurred with treatment with diosmin as monotherapy or when it was combined with linagliptin.

The glucose-6-phosphatase activity in the current study showed a significant upregulation in the diabetic group when compared with the healthy control group. This is in agreement with the results of Mahmoud et al. [30], who demonstrated that in comparison to the normal control, there was a notable increase in the activity of the gluconeogenic enzymes fructose 1, 6-bisphosphatase and glucose-6-phosphatase, as well as of hepatic glycogen phosphorylase. The activity of glucose-6-phosphatase exhibited a significant decrease in the group of diabetic animals treated with linagliptin when compared with diabetic control.

On treatment with diosmin, the activity of glucose-6-phosphatase showed a significant decrease when compared with the diabetic control. The results fit the previous results of Pari and Srinivasan [27] and Zheng et al. [31], revealing an increase in the activity of glucose-6-phosphatase in the animals with diabetes receiving diosmin. Combination therapy with both linagliptin and diosmin revealed a significant decrease when compared to the monotherapies in this study; thus, diosmin and linagliptin might have synergistic effects on the liver glucose-6-phosphatase activity.

Insulin resistance and β-cell dysfunction have both been linked to oxidative stress [32]. Pancreatic cell damage has been linked with the development of experimental DM that was induced by STZ [33]. In this case, in addition to the damaging effects of STZ on β-cells, the subsequent hyperglycemia induces the production of free radicals, which can exhaust the antioxidant defense mechanism, resulting in oxidative damage to cell membranes and increased vulnerability to LPO [34]. In our study, oxidative stress and antioxidant defense systems were detected by measuring levels of several biomarkers in the liver.

Secondary products such as MDA are used to quantify LPO indirectly [35]. Our present exploration showed a significant increase in liver LPO levels in the diabetic rats compared with the healthy ones. The results align with the results of Mohamed et al. [36]. On treatment with linagliptin, the level of MDA exhibited a significant decrease due to the amelioration of lipids in the diabetic group treated with linagliptin and thus reflected on LPO. This result is further supported by the studies of the protective potential effect of linagliptin against hepatic encephalopathy induced by thioacetamide (TAA) in rats [37]. In the current study, the diabetic groups treated with diosmin exhibited no significant effect when compared with the untreated diabetic rats. The observed results fit the results of Ağır who studied the effect of diosmin against liver damage and detected that it had no significant effect on liver LPO represented by the level of MDA [38]. However, it contrasts with another result explained in the study of Anwer who observed that the administration of diosmin showed a significant decrease in the level of MDA in Wistar rats, which suffered from liver injury [39]. The combination of diosmin and linagliptin in our study revealed no significant effect on hepatic LPO, which suggests that the effect of linagliptin on liver LPO may be decreased by the diosmin combination, but it may have been due to the short experiment time and treatment duration.

The current study revealed modulation of the liver GSH content when treated with linagliptin because of the improvement of insulin resistance, as some results state that insulin resistance is also linked to oxidative damage. According to certain research, oxidative stress is also responsible for altering intracellular signaling pathways, which leads to insulin resistance [40]. In our research, the diabetic rats treated with diosmin showed a significant increase in hepatic GSH on comparison with the untreated group. These results are supported by the detected results of Liu in his study, which investigated the diosmin effect on the ARPE-19 human retinal pigment epithelial cells exposed to high glucose and observed the increased effect of diosmin treatment at high-glucose ARPE-19 cells on the GSH concentration level [41]. On combination therapy of the diabetic rats with linagliptin and diosmin, the level of hepatic GSH content significantly increased, indicating the improvement of hepatic oxidative stress as a diabetic complication.

The current study revealed that glutathione GPx showed a significant decrease in the diabetic rats. On the other hand, the diabetic rats treated with linagliptin and/or diosmin exhibited a significant increase in the hepatic GPx enzyme activity, indicating a potent antioxidant effect of linagliptin and diosmin.

Diabetes consequences, both microvascular and cardiovascular, are heavily influenced by oxidative stress. Diabetic metabolic problems increase mitochondrial superoxide generation. The central and significant mediator of diabetic tissue damage is increased superoxide generation [42]. SOD and GST are ROS-scavenging enzymes that decrease in the case of DM.

On the other hand, the activity of GST increased due to an increase in their production when treating the diabetic rats with linagliptin as monotherapy and in combination with diosmin. On treatment with diosmin as monotherapy, there was an improvement in the GST activity and a significant increase in comparison with the diabetic control group. This result is consistent with the results of [43], which studied the alleviative efficacy of diosmin against STZ/NA-induced oxidative stress in diabetic rats.

In our current study, the generation of SOD increased significantly in the diabetic rats treated with linagliptin as it had antioxidant effects on the diabetic rats. This significant increase in SOD was also detected with diosmin monotherapy and combination therapy with linagliptin. This approves our suggestion of the effectiveness of the combination of linagliptin with diosmin in treating DM type 2 in experimental animals. This result fits with the result of Srinivasan and Pari [43], who detected a significant increase in the recovery of SOD activity due to the treatment of diabetic animals with diosmin.

IL-10, a Th2 (T helper 2) cytokine, is considered an anti-inflammatory marker suitable for detecting inflammation in DM patients. In our present investigation, the serum level of IL-10 decreased in the diabetic rats. On the other hand, by treating the diabetic animals with linagliptin and/or diosmin, the serum IL-10 level was significantly elevated; the combined effect was the most effective, reflecting that the combination treatment has the most potent anti-inflammatory effect. These results are in accordance with those observed by Arab et al., who revealed the anti-inflammatory action of diosmin [44]. The combination therapy of linagliptin with diosmin revealed a significant increase in the serum IL-10 level compared to monotherapy with linagliptin.

Adiponectin is a peptide generated from adipocytes that has anti-inflammatory and insulin sensitivity effects [45]. A nested case–control study was undertaken to determine if baseline adiponectin concentrations in plasma are independently linked with the risk of T2DM [46]. In the presently observed results, adiponectin exhibited a significant increase in expression when linagliptin was used as monotherapy for the NA/STZ-induced diabetic rats compared with the untreated diabetic control group of animals. This observation is consistent with the results of Koyama et al. when the endothelial function in patients with T2DM and coronary artery disease was studied using linagliptin and voglibose [47]. On the other hand, the treatment with diosmin also showed a significant increase in the adiponectin expression, supporting the results of Gerges et al. who demonstrated that diosmin dramatically recovers circulating adiponectin levels [48]. In the present study, the treatment with diosmin and linagliptin produced a significant increase in the adiponectin expression, assuming the improvement of insulin sensitivity because of the increasing expression of adiponectin.

Resistin belongs to the resistin-like molecule family of tissue-specific signaling molecules. It is considered to be a proinflammatory cytokine involved in linking obesity and insulin resistance in T2DM [49]. Its levels in plasma are elevated in hyperglycemic and hyper-insulinemic rodents, as well as in T2DM patients [45,49,50,51,52]. In the present study, the resistin expression gene exhibited a significant increase in the diabetic rats when compared with the healthy non-diabetic group. By treatment with linagliptin, diosmin, and both of them, the levels of resistin expression showed a significant decrease.

The serum urea and creatinine levels were significantly elevated in the NA/STZ-induced diabetic rats; these results are in accordance with many publications [53,54,55]. The treatments of the diabetic rats with diosmin, linagliptin, and both of them resulted in a significant decrease in the elevated levels of urea and creatinine. The combined effect was the most potent. These changes in the serum kidney function parameters led us to suggest that diosmin, linagliptin, and both of them not only have antihyperglycemic, anti-inflammatory, and antioxidant effects, but also improve the kidney function.

In conclusion, it is clear from our data in this study that diosmin has numerous antidiabetic effects as monotherapy or in combination with linagliptin. The combined effect of diosmin with linagliptin was the most potent on OGT, hepatic glucose-6-phosphatase activity, and serum creatinine level. The antidiabetic effect was proved by the improvement that occurred in insulin resistance and the increase in insulin sensitivity in diabetic cells, as well as amelioration of the pancreatic islets’ histological integrity and the insulin formation by the recovered β-cells. The recovery effect produced a decrease in the serum glucose levels, an increase in the insulin and C-peptide levels, and an improvement of the glucose homeostasis indexes. Linagliptin and diosmin improved oxidative stress parameters such as the LPO, SOD, GPx, GST activities and the GSH content in the livers of NA/STZ-induced diabetic rats. Linagliptin and diosmin exhibited a significant increase in anti-inflammatory markers such as IL-10. The current study data showed improvement in the gene expression of adiponectin and resistin in visceral adipose tissue that reveals the effective action on insulin resistance of NA/STZ-induced diabetic rats. Thus, it is speculated that diosmin in combination with linagliptin may be suggested as an efficient antihyperglycemic treatment for T2DM after approval of its efficacy and safety in humans. Further investigations for longer durations are necessary in the future to detect the effects on molecular mediators in the signaling pathways of insulin action, insulin secretion, inflammation, and antioxidant defense mechanisms.

## 4. Materials and Methods

### 4.1. Chemicals

NA and STZ were purchased from Sigma, MO, USA. The glucose kit was provided by Spinreact (Gerona, Spain). The insulin and C-peptide ELISA kit was purchased from MyBioSource (San Diego, CA, USA). Interleukin 10 was determined using a rat IL-10 Quantikine ELISA kit (R&D Systems, Inc., Minneapolis, MN, USA). Total ribonucleic acid (RNA) was extracted from the adipose tissue using a Qiagen tissue extraction kit (Qiagen, San Diego, CA, USA) based on the manufacturer’s instructions. The other chemicals and reagents that were employed were all highly pure and of analytical quality.

### 4.2. Experimental Animals

Adult male Wistar rats weighing 120–140 g were used in the present study to perform the experiment. The experimental animals were delivered from the Animal Facilities Center of the National Research Center in Cairo, Egypt. Before the experiment started, the rats were kept under surveillance for a week in order to rule out any prior infections. The animals were housed in cages made from polypropylene and kept at controlled room temperature (25 ± 2 °C) and humidity (55 ± 5%). The animals had access to drinking water and a standard pellet diet ad libitum. All the experimental procedures were completed in accordance with the instructions and guidelines and were approved by the Animal Ethics Committee, Beni-Suef University, Egypt (Ethical Approval number 022-463). All efforts were directed towards reducing the number and suffering of animals.

### 4.3. Induction of DM

To induce T2DM, the groups of overnight-fasted rats were injected intraperitoneally (i.p.) with NA at a dose of 60 mg/kg (dissolved in isotonic saline) 15 min after an intraperitoneal injection of STZ (60 mg/kg; dissolved in citrate buffer, pH 4.5) [56,57]. The same volume of vehicles, citrate buffer (pH 4.5) and 0.9% saline, were administered to the corresponding normal control rats.

### 4.4. Experimental Design and Blood and Tissue Sampling

Blood samples were centrifuged at 3000 RPM for 15 min and the clear non-hemolyzed supernatant sera were separated into three portions for each individual animal and kept at −20 °C until used. One gram of frozen liver tissue was homogenized in 10 mL ice-cold saline (0.9% NaCl) to yield 1% homogenate (*w*/*v*) [16]. The liver homogenate was centrifuged at 3000 RPM for 15 min. The supernatants were separated and kept in a deep freezer at −20 °C until they were used in biochemical analysis. One part of organ tissues was preserved in 70% alcohol after fixation using neutral buffered formalin for 24 h to prepare for histopathological examination [58].

Five groups, each with 6 adult male Wistar rats, were utilized in this investigation:

- Normal control group (NC). These were healthy rats that were given an equivalent volume of carboxymethyl cellulose (CMC) (1% *w*/*v*) every other day for 28 days by oral gavage.
- Diabetic control group (DC). This group consisted of diabetic rats that were given the equivalent volume of CMC (1% *w*/*v*) by oral gavage every other day for 28 days.
- Diabetic group treated with linagliptin (D + LIN). This group consisted of diabetic rats, but they were treated with linagliptin at a dose of 1 mg/kg (dissolved in 1% CMC) every other day for 28 days by oral gavage [59].
- Diabetic group treated with diosmin (D + DIO). In this group, diabetic rats were treated with diosmin at a dose of 10 mg/kg (dissolved in 1% CMC) every other day for 28 days by oral gavage [60].
- Diabetic group treated with both linagliptin and diosmin (D + LIN + DIO). The rats in this group were diabetic rats that were treated with linagliptin at a dose of 1 mg/kg and diosmin at a dose of 10 mg/kg every other day for 28 days by oral gavage.

### 4.5. Blood Sampling and Tissue Sampling

At the end of the 4th week of the treatment period, the rats were anesthetized with ethyl ether, and from the jugular vein, blood samples were taken in Gel & Clot activator tubes. Serum was separated by centrifugation at 3000 RPM for 15 min, and clear non-hemolyzed supernatant sera were separated and kept at −20 °C until used. After decapitation and dissection, organ tissue specimens, liver, and pancreas were excised for histological and biochemical analyses.

### 4.6. Biochemical Analysis

To assess the antihyperglycemic effects, OGTT was performed. OGTT was performed by measuring the blood glucose of the rats at time intervals of 0, 60, 120 and 180 min after oral uptake of the glucose dose (3 g/kg) according to Ibrahim et al. [61] (using a glucometer and Gluco Dr.auto strips (All Medicus, Co., Ltd., Anyang-si, Republic of Korea)). The day before the sacrifice, blood samples were obtained by puncturing the lateral tail vein following a 10–12 h fast.

To assess the effect on insulin formation and insulin secretory response of β-cells, serum insulin and C-peptide levels were detected. Using an ELISA kit from MyBioSource (San Diego, CA, USA), serum insulin and C-peptide concentrations were measured in accordance with the manufacturer’s instructions.

Hepatic glycogen content, which is another glycemic indicator, was determined according to Seifter et al. [62]. To assess the effects on hepatic glucose output, hepatic glucose-6-phosphatase and glycogen phosphorylase activities were determined. Hepatic glucose-6-phosphatase activity was estimated based on the procedure of Kabir and Begum [63]. Hepatic glycogen phosphorylase activity was detected based on the method of Stalmans and Hers [64] using reagents prepared in the laboratory.

The homeostasis model assessment (HOMA) indices were evaluated to scrutinize the effects on insulin action and β-cell function. HOMA-insulin resistance (HOMA-IR), HOMA-insulin sensitivity (HOMA-IS), and HOMA-β-cell function (HOMA-β) were calculated according to the following equations [65,66,67]:

HOMA-IR = (fasting insulin [µIU/mL] × fasting glucose [mg/dL])/405

HOMA-IS = 10,000/(fasting insulin [µIU/mL] × fasting glucose [mg/dL])

HOMA-β cell function = (20 × fasting insulin [µIU/mL])/(fasting glucose [mg/dL] − 3.5)

Serum urea and creatinine were detected to assess the effects on the kidney function. Serum urea was determined by a method using kits obtained from Randox Laboratories Company (Crumlin, UK) according to Patton and Crouch [68]. The serum creatinine level was determined by a method using kits obtained from Randox Laboratories Company (UK) according to Bartels and Bohmer [69].

IL-10, which is an anti-inflammatory cytokine, was determined using a rat ELISA kit according to the guidelines and instructions of the manufacturer (Bio-Techne, Minneapolis, MN, USA). This assay depends on the quantitative sandwich enzyme-linked immunoassay technique.

### 4.7. RNA Isolation and Quantitative Real-Time Polymerase Chain Reaction (qRT-PCR)

Total RNA was extracted from the adipose tissue using a Qiagen tissue extraction kit in accordance with the manufacturer’s instructions (Qiagen, Germantown, MD, USA). Quantitative real-time polymerase chain reaction (qRT-PCR) amplification and analysis were performed using an Applied Biosystem running software version 3.1. (Applied Biosystems, Waltham, MA, USA).

Complementary DNA (cDNA) was synthesized according to the manufacturer’s protocol (Thermo Fisher Scientific, Waltham, MA, USA). A 2 µg of cDNA template, SYBR^®^-Green Master Mix (Thermo Fisher Scientific, Waltham, MA, USA), and the appropriate primers were used to conduct qRT-PCR analyses, which were conducted in triplicate. The 2^−ΔΔCt^ method [70] was used for calculating the relative gene expression levels. The β-actin gene was amplified as an internal control. The primer sequences used included the following: for adiponectin F: 5′-AATCCTGCCCAGTCATGAAG-3′ and R: 5′-CATCTCCTGGGTCACCCTTA-3′ [71]; for resistin F: 5′-GGGAGTTGTGCCCTGCT-3′; R: 5′-CAGCACTCGGAGGGCAA-3′ [72].

### 4.8. Determination of Oxidative Stress and Anti-Oxidant Defense Parameters

The technique of Marklund and Marklund [73] was applied to measure the liver SOD enzyme activity. In short, pyrogallol quickly underwent auto-oxidation in an aqueous solution to generate a yellow color that was detectable at 430 nm. A 0.1 mL of Tris buffer (pH 8) was added to one mL of homogenate supernatant. Then, 0.05 mL of pyrogallol were added. The initial absorbance was read immediately and read again 10 min after adding pyrogallol. The control consisted of 0.1 mL Tris buffer and one mL distilled water; then, 0.05 mL of pyrogallol were added. The enzyme activity and the inhibition of the yellow color that emerged at 430 nm were computed. An enzyme unit was defined as the quantity of the enzyme that resulted in a 50% suppression of the extinction changes in 1 min as compared to the control.

Formation of thiobarbituric acid-reacting substances (TBARS), low-molecular-weight end products (mainly MDA) that are formed during the decomposition of lipid peroxidation products, estimates the amount of lipid peroxidation (LPO). MDA was assayed based on the procedure described by Preuss et al. [74]. The protein was precipitated by mixing 1 mL of the liver homogenate with 0.15 mL of 76% trichloroacetic acid (TCA). Then, as a color-developing agent, 0.35 mL of thiobarbituric acid (TBA) were added to the separated supernatant. After being incubated for 30 min at 80 °C in a water bath, the formed faint pink color was detected at 532 nm. MDA, also known as 1,1,3,3-tetramethoxypropane, was employed as the standard.

The amount of GSH was measured using the technique of Beutler [75]. In brief, Ellman’s reagent (as a color-developing agent), 0.5 mL DTNB [5,5′-dithiobis (2-nitrobenzoic acid)], and phosphate buffer solution (pH 7) were added to the supernatant following protein precipitation in order to quantify GSH. The sample and standard’s produced yellow color was measured at 412 nm in comparison to the blank, and the GSH concentration in the sample was calculated.

The activity of GPx was estimated using the suggested method of Matkovics et al. [76]. In this method, the residual GSH after a certain time from the known concentration of GSH added to the sample was measured photometrically at 412 nm by reacting with DTNB. Then, the reduced form of GSH was converted to the oxidized form (GSSG), and the total amount was calculated. Briefly, 350 μL Tris buffer (pH 7.6), 50 μL GSH solution (2 mM), and 50 μL H_2_O_2_ (3.38 mM) were placed in a Wasserman tube, and 50 μL homogenate supernatant was added. Following ten minutes of incubation, the previously described method [75] for determining GSH at 430 nm was used to measure the residual GSH content. The blank test was made by adding 100 μL of distilled water in place of 50 μL of the sample and 50 μL of the GSH solution, and the standard test was performed by adding 50 μL of distilled water in place of 50 μL of the sample. Following the identification of the sample’s residual GSH content, the GSH converted to the oxidized form (GSSG), and the enzyme activity can be computed.

GST activity was determined based on the chemical method described in a previous publication [77]. In brief, 250 µL of 4 mM 1-chloro-2,4-dinitrobenzene (CDNB) was introduced into a Wasserman tube that had 250 µL of the sample, 250 µL of the GSH solution (4 mM), and 250 µL of phosphate buffer (pH 7.3). After 10 min of incubation at 25 °C, the developed color was measured.

### 4.9. Histological and Immunohistochemical Investigations

The pancreas was quickly removed after dissection, chopped into tiny three mm^3^ pieces, and then preserved for 24 h in 10% neutral buffered formalin. The tissues were washed and dehydrated in two changes of 100% ethyl alcohol for 30 min each after the excess fixative removed. This was followed by 45 min each of increasing grades of ethyl alcohol: 70%, 80%, 90%, and 95%. After that, two changes of xylene were used to clear the tissues for thirty minutes each. After being impregnated with paraplast plus (three changes) for three hours at 60 degrees Celsius, the tissues were implanted in paraplast plus. Sections 4 to 5 µm thick were created using a microtome, and for histological examination, they were then stained with hematoxylin and eosin [78].

For immunohistochemical investigation, sections 5–6 µm thick that were mounted on positive slides and stained using the streptavidin–biotin–peroxidase staining method were used for anti-insulin immunolocalization [17,79]. To enable comparison of the immunostaining outcomes across the different experimental groups, each section was incubated with the same quantity of primary and secondary antibodies for the same length of time and under the same circumstances [16]. Following immunohistochemical staining, light microscopy was used to examine the stained sections. A positive reaction was shown by a brown hue. Insulin granules’ integrated density and area percentage as indicated by brown color were evaluated using the ImageJ (1.54d version) (http://imagej.org, accessed on 13 September 2023; Wayne Rasband and Contributors, National Institutes of Health, Bethesda, MD, USA).

### 4.10. Statistical Analysis

The average ± standard deviation (SD) was used to express the data. SPSS version 20 statistical analysis was used to analyze the data using one-way analysis of variance (ANOVA) followed by Tukey’s test to compare various groups with each other. Significant differences were found for values of *p* < 0.05.

## Figures and Tables

**Table 1:** Effect of linagliptin and diosmin on OGT in NA/STZ-induced diabetic rats.

	Time (Min)	0 min	60 min	120 min	180 min
Groups					
NC		77.125 ± 5.21 ^a^	127.88 ± 19.75 ^a^	111.5 ± 5.68 ^a^	81.00 ± 7.89 ^a^
DC		438.70± 36.21 ^c^	598 ± 46.47 ^d^	560 ± 80.67 ^d^	498.00 ± 49.83 ^c^
D + LIN		242.25 ± 65.8 ^ab^	377.5 ± 88.18 ^bc^	287 ± 62.09 ^bc^	319.00 ± 76.40 ^b^
D + DIO		366.75 ± 76.49 ^bc^	502.25 ± 65.54 ^cd^	405.25 ± 56.05 ^c^	418.67 ± 87.07 ^bc^
D + LIN + DIO		141.25 ± 35.15 ^a^	255 ± 55.6 ^ab^	169.75 ± 56.83 ^ab^	161.25 ± 30.99 ^a^

Data expressed as the means ± SE. Means which do not share the same superscript symbols (a, b, c, and d) within the same column, are significantly different at *p* < 0.05. F-probability: *p* < 0.001. NC, DC, D + LIN, D + DIO, and D + LIN + DIO refer to the normal control group, the diabetic control group, the diabetic group treated with linagliptin, the diabetic group treated with diosmin, and the diabetic group treated with linagliptin and diosmin.

## Data Availability

All data are available from the corresponding author upon reasonable request.

## Abbreviations

DM: diabetes mellitus; DS: diosmin; DPP4: dipeptidyl peptidase-4;WHO: World Health Organization; CMC: carboxy methyl cellulose; ELISA: sandwich enzyme-linked immunosorbent assay; GPx: glutathione peroxidase; GST: glutathione-S-transferase; IL: islet of Langerhans; H&E: hematoxylin and eosin; HOMA-IR: homeostatic model assessment-insulin resistance; HOMA-IS: homeostatic model assessment-insulin sensitivity; HOMA-β cell function: homeostatic model assessment-β cell function; GSH: reduced glutathione; i.p.: intraperitoneal; IR: insulin resistance; IS: insulin sensitivity; LPO: lipid peroxidation; MDA: malondialdehyde; mRNA: messenger RNA; OGTT: oral glucose tolerance test; RNA: ribonucleic acid; qRT-PCR: quantitative real-time polymerase chain reaction; SOD: superoxide dismutase; STZ: streptozotocin; T2DM: type 2 DM; DTNB: 5,5′dithiobis-2-nitrobenzoic acid; CDNB: 1-choloro-2,4-dinitrobenzene; IL-10: interleukin 10; SE: standard error.
